# Basement Membrane of Tissue Engineered Extracellular Matrix Scaffolds Modulates Rapid Human Endothelial Cell Recellularization and Promote Quiescent Behavior After Monolayer Formation

**DOI:** 10.3389/fbioe.2022.903907

**Published:** 2022-08-02

**Authors:** Manuela Lopera Higuita, Nicholas A. Shortreed, Surendra Dasari, Leigh G. Griffiths

**Affiliations:** Mayo Clinic, Rochester, MN, United States

**Keywords:** extracellular matrix, tissue engineering, cardiovascular diseases, small diameter vessels, cell behavior

## Abstract

Off-the-shelf small diameter vascular grafts are an attractive alternative to eliminate the shortcomings of autologous tissues for vascular grafting. Bovine saphenous vein (SV) extracellular matrix (ECM) scaffolds are potentially ideal small diameter vascular grafts, due to their inherent architecture and signaling molecules capable of driving repopulating cell behavior and regeneration. However, harnessing this potential is predicated on the ability of the scaffold generation technique to maintain the delicate structure, composition, and associated functions of native vascular ECM. Previous de-cellularization methods have been uniformly demonstrated to disrupt the delicate basement membrane components of native vascular ECM. The antigen removal (AR) tissue processing method utilizes the protein chemistry principle of differential solubility to achieve a step-wise removal of antigens with similar physiochemical properties. Briefly, the cellular components of SV are permeabilized and the actomyosin crossbridges are relaxed, followed by lipophilic antigen removal, sarcomeric disassembly, hydrophilic antigen removal, nuclease digestion, and washout. Here, we demonstrate that bovine SV ECM scaffolds generated using the novel AR approach results in the retention of native basement membrane protein structure, composition (e.g., Collagen IV and laminin), and associated cell modulatory function. Presence of basement membrane proteins in AR vascular ECM scaffolds increases the rate of endothelial cell monolayer formation by enhancing cell migration and proliferation. Following monolayer formation, basement membrane proteins promote appropriate formation of adherence junction and apicobasal polarization, increasing the secretion of nitric oxide, and driving repopulating endothelial cells toward a quiescent phenotype. We conclude that the presence of an intact native vascular basement membrane in the AR SV ECM scaffolds modulates human endothelial cell quiescent monolayer formation which is essential for vessel homeostasis.

## 1 Introduction

Diseases of small diameter blood vessels (i.e., < 6 mm) such as coronary artery diseases and peripheral vascular diseases affect over 25 million people in the United States (Fryar et al., 2012; Benjamin et al., 2019). While a wide array of treatments have been evaluated, the use of autologous tissues for vascular bypass remains the standard of care for small diameter vessel diseases, with over 4.2 million people undergoing vascular grafting every year (Slovut and Lipsitz, 2012; Alexander and Smith, 2016). However, lack of suitable autologous grafts due to pre-existing vascular diseases deprive approximately one-third of patients from receiving life-saving vascular grafting (Hillis et al., 2011; Popovic et al., 2013; Taggart, 2013; de Vries et al., 2016; McKavanagh et al., 2017; Tranbaugh et al., 2017). Additionally, for patients with suitable vasculature, autologous tissue harvesting results in increased morbidity and potential for donor site complications (Hillis et al., 2012). The development of an off-the-shelf small diameter vascular graft has the potential to overcome the challenges associated with autologous vessel harvest, providing a solution with sufficient length and unlimited availability applicable for the treatment of vascular diseases in all patients.

Acellular extracellular matrix (ECM) scaffolds derived from xenogeneic vascular tissue have the potential to serve as an ideal source for off-the-shelf vascular grafts. For vascular tissues, in addition to providing structural support, the ECM contains growth factors, proteases, cytokines, proteoglycans, collagens, laminin, fibronectin fibrils, and other biologically active molecules, each of which have the potential to influence repopulating endothelial cell proliferation, differentiation, migration, and apoptosis (Faulk et al., 2014a). The effects of individual native ECM proteins, particularly vascular basement membrane components, have been extensively researched and shown to modulate advantageous endothelial cell behaviors, with the potential to mediate rapid monolayer formation and drive endothelial cells toward a quiescent phenotype (Morrissey and Sherwood, 2015). Laminin for instance, has been demonstrated to mediate human endothelial cell differentiation into capillary-like structures, while collagen IV (Col IV) has been demonstrated to influence endothelial cell adhesion and migration (Xing et al., 2021). While independent individual effects of important basement membrane proteins on endothelial cell behavior have been documented by previous *in vitro* studies, such single molecule studies fail to recapitulate the structural organization and compositional complexity of native vascular basement membranes. Determining the combined effect of these complex ECM factors on endothelial cell behavior in intact ECM scaffolds has not been possible due to the inability of de-cellularization methods to retain native ECM protein structure, composition, and function (Crapo et al., 2011). In particular, disruption of delicate endothelial basement membrane components (e.g., collagen IV, laminin) is very common with de-cellularization approaches, regardless of specific chemicals employed (Faulk et al., 2014b). Additionally, the disruption of ECM proteins caused by tissue processing methods have further implications, as modified ECM proteins have the potential to negatively modulate endothelial cell behavior and lead to cellular dysfunction possibly causing inflammatory syndromes, intimal hyperplasia, thrombosis (Huang and Greenspan, 2012; Lee et al., 2014).

The purpose of this article is to determine the combined effect of ECM basement membrane factors on the endothelial cell behavior by utilizing antigen-removed ECM vascular scaffolds. Antigen removal (AR), unlike the common de-cellularization technique, is a tissue processing method shown to retain the composition and function of ECM proteins, while significantly reducing the antigenic content from xenogeneic tissues (Dalgliesh et al., 2018; Lopera Higuita and Griffiths, 2020a). Specifically, when applied to a small diameter bovine saphenous vein (SV, <3.5 mm diameter), AR tissue processing resulted in the elimination of 98.5% of the tissue antigenic components while retaining the predominant structural, functional, and compositional characteristics of the ECM (Lopera Higuita and Griffiths, 2020a). The retention of the ECM proteins was determined by the extensive characterization of antigen- removed ECM scaffolds in the previous work, where the mechanical properties of the acellular scaffold and the content of elastin, desmosine, collagen, and pyridinaline were all retained compared to untreated tissues (Lopera Higuita and Griffiths, 2020a).

Furthermore, retention of important endothelial basement membrane proteins by the AR tissue processing method suggests that AR-SV scaffolds are an ideal testing material to determine the drivers of endothelial behavior in native ECM. The anisotropic organization of SV (i.e., luminal surface of the vessel, which is lined with basement membrane proteins (BM) and abluminal surface of the vessel, which is mainly composed of loose structural non-basement membrane proteins (e.g., collagen I) (NBM)) allows for the comparison of endothelial cell response in the presence versus absence of basement membrane components, in otherwise identical scaffolds (i.e., processed *via* the same tissue processing method). We hypothesize the presence of basement membrane proteins in the lumen side of the AR-SV vascular graft (i.e., BM side seeding) will: 1) increase the rate at which seeded endothelial cells form a monolayer and 2) drive the quiescence cell behavior once a confluent monolayer is formed, when compared to the endothelial cell response in the absence of such proteins (i.e., NBM side seeding).

## 2 Materials and Methods

All chemicals were purchased from Sigma-Aldrich (St. Louis, MO) unless otherwise stated. All experiments were performed with n = 6 replicates per group, unless otherwise stated.

### 2.1 Tissue Harvest

Fresh bovine saphenous veins (SV) were harvested and processed as previously stated (Lopera Higuita et al., 2021).

### 2.2 Antigen Removal

All ECM scaffold generation steps were performed in 4 ml of solution at RT, 150 rpm, and changed twice daily, unless otherwise stated. Previously frozen SV were thawed, planarized by incising the vessel longitudinally and cut into discs using 14 mm biopsy punches, unless otherwise stated. All chemicals were added to a base buffer solution (0.5 mM Pefabloc, 1% v/v antibiotic antimycotic solution (AAS) in 10 mM Tris–HCl (pH 8.0). Antigen removal (AR) samples (scaffolds) underwent sarcomeric relaxation (3% amidosulfobetaine-16 (ASB-16), 120 mM potassium chloride (KCl), 4 mM MgCl_2_, 4 mM EDTA, 5.88 mM Na-ATP, 10 mM 2,3 Butanedione monoxime (BDM), 0.5 mM Pefabloc, and 1% AAS in 10 mM Tris–HCl, pH 7.6) twice for 30 min each. Relaxed veins were incubated in a lipophilic protein solubilization solution (3% ASB-16 in 100 mM dithiothreitol (DTT), 2 mM MgCl_2_, and 600 mM KCl, in the base buffer) for 48 h. AR-scaffolds underwent sarcomeric disassembly by washing in the base buffer twice for 15 min, followed by incubation in 50 nM Latranculin B (Cayman Chemical, Ann Arbor, MI) in the base buffer for 2 h; washed again with the base buffer twice for 15 min and incubated for 2 h in 0.6 M KCl in the base buffer; washed a final time with the base buffer twice for 15 min and incubated 2 h in 1 M potassium iodine (KI) in base buffer, followed by overnight incubation in the base buffer alone. The KCl and KI steps were repeated the next day, followed by overnight incubation in the base buffer. Scaffolds were then placed in the lipophilic protein solubilization solution for 48 h, followed by 24 h incubation in the hydrophilic protein removal solution (100 mM dithiothreitol (DTT), 2 mM MgCl_2_ and 600 mM KCl, in base buffer). Subsequently, scaffolds underwent 24 h of nuclease digestion (10 Kunitz units/mL DNase I, 15 Kunitz units/mL RNase A, 0.5 mM Pefabloc, 1% AAS, 0.15 M NaCl, 5 mM MgCl_2_–6H_2_O in 10 mM Tris–HCl, pH 7.6) and 96 h of washout in the base buffer at 4°C (Supplementary Figure S1).

### 2.3 EC Recellularization

Human aortic endothelial cells (hAECs), GFP-labeled hAECs (GFP-hAECs), human umbilical vein cells (HUVECs), and GFP-labeled HUVECS (GFP-HUVECs, all cells from Angio-Proteomie, Boston, MA) were expanded to P4, unless otherwise stated, in a T-25 flask coated with quick coating solution (Angio-Proteomie) at 37^°^C, 5% CO_2_. Cells were cultured in 7 ml of the endothelial cell growth medium (Lonza, Allendale, NJ) with the solution changed at 24 h after thawing once every 3 days. Cells were lifted using Accutase^®^ solution, hAECs were re-seeded on scaffolds at a cell seeding density of 450 cells/mm^2^ and HUVECs were re-seeded at a cell density of 600 cells/mm^2^, unless stated otherwise and cultured under the same conditions. Before cell seeding, all the scaffolds were incubated in 1% AAS in endothelial cell media overnight.

### 2.4 Endothelial Cell Migration Plus Proliferation Assay

Endothelial cell migration and proliferation assay was performed by seeding hAECs as a 3.5 mm disc of cells in the center of 1 × 1 cm square scaffolds in 24-well plates. Scaffolds were generated using 6 ml of the corresponding AR solutions. Scaffolds were sutured on to high temperature foam to allow the cell-seeded scaffolds to float in an inverted orientation (i.e., cell-seeded side of the scaffold facing the bottom of the plate) on top of the culture media. The high temperature foam also resisted the effect of cell traction or scaffold recoil on the scaffold during culture, thereby keeping the scaffolds planarized throughout the experiment. Additionally, these culture conditions allowed for inverted microscopy imaging of GFP-labeled cells at multiple time points without disrupting the scaffolds or seeded cells. A 3.5 mm diameter glass cylinder was used to seed GFP-hAECs in a circular shape in the center of the scaffold. Cells were allowed to adhere for 6 h in 50 µL of cell culture media. Following adhesion, the glass cylinder was removed, 500 µL of cell media was added, and cells were monitored using inverted microscopy (Nikon Eclipse Ts2R, Minato City, Tokyo, Japan) at days 1, 4, 8, 12, 16, and 20, with the culture media being changed every other days. To obtain images of the entire scaffolds, 20–30 images were taken from each scaffold at 4 x and arranged using the microscope stitch function. The combined effect of hAEC migration and proliferation was estimated using the non-directional mean migratory distance. This was calculated by measuring the total area covered by cells, fitting a circle of equivalent area, and extracting its radius using MATLAB (MATLAB^®^, The MathWorks, Inc., Natick, MA).

### 2.5 Endothelial Cell Migration Assay (Proliferation Inhibited With Mytomiocin)

For the endothelial cell migration assay, 1 × 1 cm scaffolds were generated using 6 ml of the corresponding AR solutions. Scaffolds were sutured on to high temperature foam and the cell culture was undertaken in 24-well plates. A glass cylinder 3.5 mm in diameter was used to seed the GFP-hAECs in a circular shape. The cells were allowed to adhere for 6 h in 50 µL of the cell media. After adhesion, the cells were unconfined and treated with 1 μg/ml of proliferator inhibitor (Mitomycin C) in 300 µL of the media for 48 h. Following 48 h of incubation, cell were imaged using inverted microscopy (Nikon Eclipse Ts2R, Minato City, Tokyo, Japan) at days 3, 6, and 10 post seeding. Migration was determined by the mean migratory distance obtained as described in Section 2.4.

### 2.6 Endothelial Cell Proliferation Assay (*via* FUCCI Staining)

For the endothelial cell proliferation assay, 1 × 1 cm scaffolds were generated using 6 ml of the corresponding AR solutions. The scaffolds were sutured on to high temperature foam, to avoid scaffold recoil, and the cell culture was undertaken in 24-well plates. The hAEC cells were transfected using the Premo FUCCI Cell Cycle Sensor (ThermoFisher Scientific, Waltham, MA) following the manufacturer’s protocols. Briefly, early passage (P2) hAECs were seeded on to six well tissue culture plastic coated with a quick coating solution (Angio-Proteomie) at a 50% confluency rate, in 3 ml of media and allowed to adhere overnight. After cell adhesion, hAECs were treated with 120 particles per cell (PPC) of Premo FUCCI in 500 µL of the cell media overnight. After transfection, cells were detached and seeded onto scaffolds using a 12 mm internal diameter stainless steel cylinder at a density of 250 cell/mm^2^ (i.e., ∼50% confluency) in 500 µL of the media and allowed to adhere overnight. After 12 h of post-seeding, the proliferating cells were counted using inverted microscopy (Nikon Eclipse Ts2R, Minato City, Tokyo, Japan).

### 2.7 Histology

Sample sections were stained with Hematoxylin and Eosin (H&E), Verhoeff van Gieson staining (VVG), and Picro–Sirius Red (PSR). Collagen birefringence was measured from PSR slides using polarized light and the percentage area of collagen polarization was calculated. For immunofluorescence staining of adherence junctions and cell polarization, antigen retrieval was performed *via* a heat-mediated sodium citrate buffer (10 mM Sodium citrate, 0.05% Tween 20, pH 6.0) and non-specific binding was blocked. For adherence junction staining, slides were incubated in rabbit anti–VE cadherin and mouse anti-β-catenin primary antibodies (11 μg/ml, 5 μg/ml respectively, Abcam, Cambridge, MA). For cell polarization, slides were incubated with mouse anti-podocalyxin (PODXL) and rabbit anti-collagen IV antibody (1:100, 1:200, respectively, Abcam). Fluorescent anti-mouse secondary antibody (1:200, Abcam) tagged with Alexa Fluor 546, anti-rabbit secondary antibody tagged with Alexa Fluor 647 (1:200, Abcam), and DAPI (ProLongTM Gold antifade reagent with DAPI, Invitrogen) were used for visualization.

For bovine laminin and collagen IV staining, sections were deparaffinized and antigen retrieval was performed *via* proteinase K (DAKO, Carpinteria, CA) at room temperature. Bovine laminin was stained using a rabbit anti-laminin antibody (1:20, Invitrogen, Carlsbad, CA). Collagen IV was stained using a rabbit anti-collagen IV antibody (1:200, Abcam). The fluorescent anti-rabbit secondary antibody tagged with Alexa Fluor 647 (1:200, Abcam) and DAPI (ProLongTM Gold antifade reagent with DAPI, Invitrogen) was used for visualization. For all stains, the slides were imaged using a Nikon Eclipse E600 microscope (Nikon, Minato City, Tokyo, Japan) and digital images were collected.

### 2.8 Human Laminin Production by Seeded Human Cells

For human laminin quantification, scaffolds seeded with hAECs at 100% confluency were fixed in 10% buffered formalin at day 4, embedded in paraffin, and 4 µm sections mounted on glass slides. Sections were de-paraffinized and antigen retrieval was performed *via* proteinase K (DAKO). Samples were stained using the rabbit anti-laminin antibody (1:200, Abcam). The fluorescent anti-rabbit secondary antibody tagged with Alexa Fluor 647 (1:200, Abcam) and DAPI (ProLongTM Gold antifade reagent with DAPI, Invitrogen) was used for visualization. Slides were imaged using a Nikon Eclipse E600 microscope (Nikon, Minato City, Tokyo, Japan) and the digital images were collected. Human laminin intensity per cell was measured for all cells in n = 6 scaffolds *via* NIS-Elements software (Nikon, Tokyo, Japan).

### 2.9 Endothelial Cell Activation/Quiescence Assessment by RNA-Sequencing

For cell activation/quiescence assessment by RNA-sequencing, hAECs were seeded at 100% confluency onto the basement membrane side of AR scaffolds (area of 1.9 cm^2^) or seeded directly into the wells of a tissue culture plate (area of 1.9 cm^2^—24-well plate). All cells were seeded at the same initial time point, after 6 h the cells seeded onto tissue culture plastic were treated overnight with either 1 µM simvastatin, 5 ng/ml TNFα, or left untreated (Control). After an overnight treatment or incubation, RNA was extracted *via* a Qiagen RNeasy mini kit, according to the manufacturer’s directions. Total RNA concentration and quality were determined using Qubit fluorometry (Invitrogen). Sequencing libraries were prepared using the Illumina TruSeq^®^ RNA Stranded Kit (Illumina, San Diego, CA) following the manufacturer’s instructions. The concentration and purity of the prepared libraries were checked using the Agilent TapeStation D1000 (Agilent Technologies, Santa Clara, CA). Libraries were sequenced at up to eight samples per lane following Illumina’s standard protocol using the Illumina cBot and HiSeq 3,000/4000 PE Cluster Kit (Illumina, San Diego, CA). The flow cells were sequenced as 100 × 2 paired end reads on an Illumina HiSeq 4,000 using the HiSeq Control Software HD 3.4.0.38 collection software (Illumina, San Diego, CA). Base-calling was performed using Illumina’s RTA version 2.7.7 (Illumina, San Diego, CA).

### 2.10 Bioinformatics Analysis of RNA-Seq Data

Raw read data were processed using a previously published MAPR-Seq pipeline and generate gene-wise read count data (Kalari et al., 2014). In brief, the sequencing data were quality controlled using the FASTQC software. Read data were aligned against the human reference genome (GRCh38) using a STAR aligner (Dobin et al., 2013). Gene expression counts were obtained using HTSeq software configured to use RefSeq human transcriptome (version 78) as the reference transcriptome (Anders et al., 2015). Gene-wise read counts observed in each sample were processed using the edgeR software for obtaining differentially expressed genes between any two experimental groups (Robinson et al., 2010). The software was configured to remove any genes with <100 counts in all samples across both groups. Read count data for the remaining genes were normalized using trimmed means of the M-values method. Gene-wise differential expression between the groups was tested using a generalized linear model configured to use negative binomial distribution. Differential expression *p*-values were corrected using the Benjamini–Hochberg method. Genes with an adjusted *p*-value <0.05 and an absolute log2 fold change ≥2.0 were considered as significantly differentially expressed and considered for further analyses.

Differential expression data of each pair-wise comparison were subjected to pathway analyses using two different methods. First, we computed the rank value of each gene using -1*log2 (*p*-value)*sign (fold change). The (gene, rank) pairs of each pair-wise comparison were subjected to gene set enrichment analyses using the PreRanked method encoded in the GSEA software, which was configured to use KEGG, REACTOME, HALLMARK, and BIOCARTA gene set databases for testing the pathway enrichment. Independently, genes that were statistically, significantly, and differentially expressed were utilized for the pathway analysis using the Webgestalt software (Liao et al., 2019). Gene sets or pathways with an adjusted *p*-value of <0.05 were considered to be statistically significant.

Z-scores for each gene were calculated and then subsequently plotted in a heatmap *via* the web-based program Heatmapper (Babicki et al., 2016). Hierarchial clustering was performed for each heatmap based on average linkage measurements.

An independent unsupervised t-distributed stochastic neighbor embedding (t-SNE) analysis was performed by ranking the entire gene expression dataset by decreasing the order of their expression coefficient of variation metric. Top ranking 1,000 genes were utilized for the t-SNE analysis. A list of (gene, eigenvalues) associated with the first two principal components (PCs) were extracted for further analysis. All gene expression analyses were performed using R statistical programming language (version 4.1).

### 2.11 Statistical Analysis

All data were analyzed for outliers using the ROUT method with Q = 1%. Migration plus proliferation data were analyzed using repeated measure 2-way ANOVA with Geisser-Greenhouse and Sidak correction. Migration-only data were analyzed using repeated measure 3-way ANOVA with the Geisser-Greenhouse and Tukey correction. All other data were analyzed with the Mann–Whitney test. All data are expressed as median ± interquartile range (IQR). Statistical significance is defined at *p* < 0.05.

## 3 Results

### 3.1 Antigen Removal Preserves Native Saphenous Veins Composition and Basement Membrane Integrity

**FIGURE 1:**
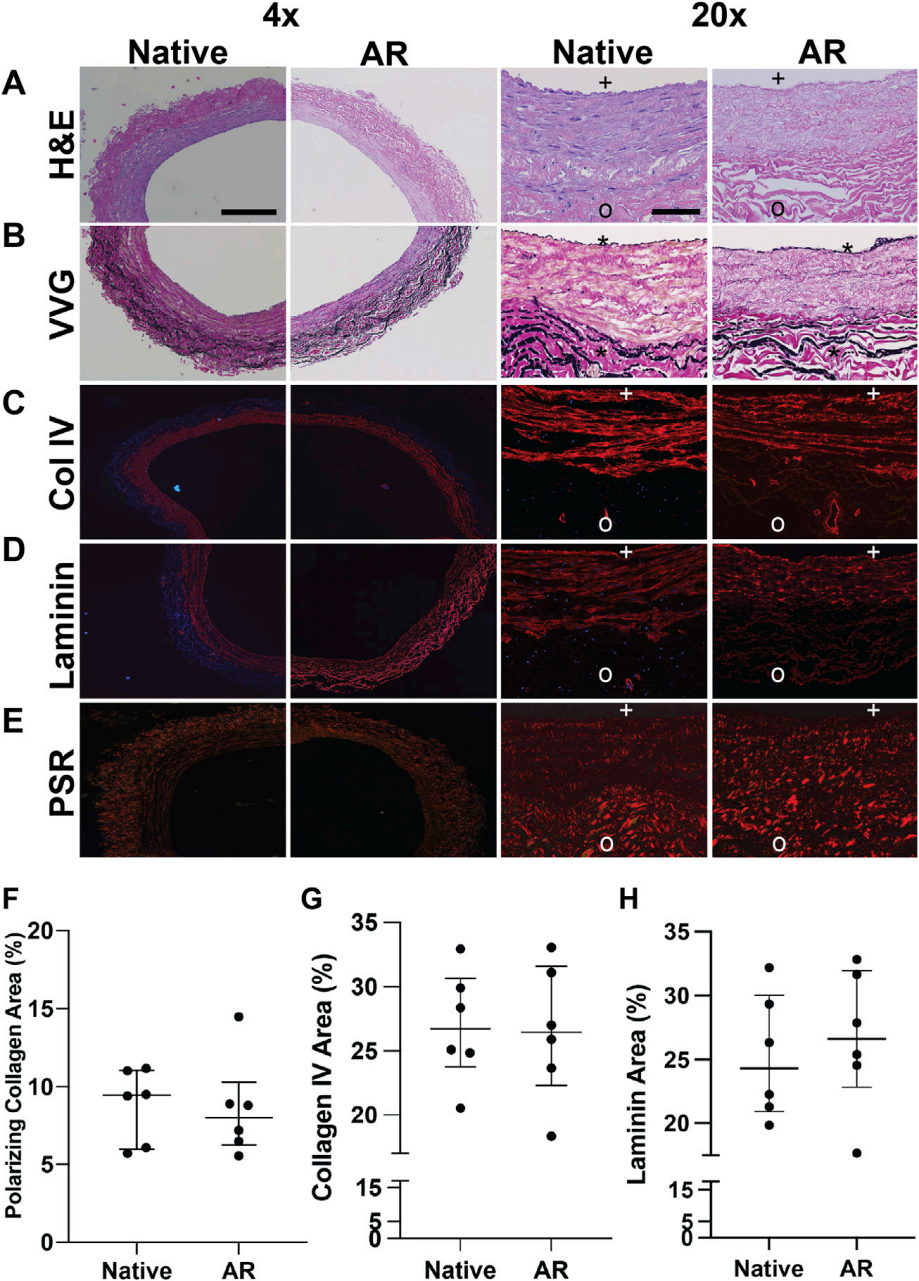
Macromolecular structure and content retention in AR–SV ECM scaffolds. Basement membrane proteins are present in the basement membrane side (BM) of the vessel (marked with + ), and are completely absent in the non-basement membrane (NBM) side (marked with o). H&E images of SV segments demonstrate complete acellularity of AR-scaffolds, while preserving the native collagen content and organization **(A)**. Elastin content and organization assessed *via* Verhoeff van Geison staining (VVG) demonstrate the retention of native SV internal (*) and external elastic laminae in AR-scaffolds **(B)**. Content and organization of basement membrane proteins (Col IV **(C)** and laminin **(D)**) in the basement membrane side of AR-SV ECM scaffolds were unchanged when compared to native tissue. Collagen alignment (PSR and polarized light) and immunofluorescence staining demonstrates the capability of AR tissue processing method of retaining the native SV collagen macromolecular structure and organization **(E)** as assessed by percent birefringence using PSR polarized light **(F)**, percent collagen IV **(G)**, and laminin **(H)**. 4 x images scale bar 200 µm. 20 x images scale bar 50 µm.

Preservation of the native SV structural (e.g., Col I) and basement membrane proteins (e.g., Laminin, Col IV) organization, content, and macromolecular integrity in AR-SV ECM scaffolds was assessed using histology, polarized light microscopy, and immunofluorescence. H&E stained sections demonstrate that AR scaffolds are completely acellular, while native ECM morphology is well preserved both in the luminal and abluminal regions (Figure 1A). VVG-stained sections demonstrate the preservation of native SV elastin and collagen content in AR scaffolds. Elastin fibril organization is preserved, as evidenced by the intact internal and external elastic laminae in AR scaffolds (Figure 1B). Immunofluorescent staining of basement membrane proteins (i.e., Col IV and laminin) demonstrated the retention of native basement membrane component content and organization on the luminal surface of AR-scaffolds (Figures 1C,D). Collagen polarization of PSR-stained sections under polarized light demonstrated the retention of native SV collagen birefringence in AR scaffolds (Figure 1E). Quantification of the collagen birefringence [7.99% (6.24–10.3)] area (Figure 1F), collagen IV [26.46% (22.33–31.6)] area (Figure 1G), and laminin [26.63% (22.8–31.95) area (Figure 1H) remained unchanged when compared to the native SV (collagen birefringence: 9.46% [5.98–11.07] area, *p* > 0.999, collagen IV: 26.73% [23.78–30.66] area, *p* > 0.999, laminin: 26.34% [21.8–30.76], *p* > 0.999).

### 3.2 Presence of Basement Membrane Proteins Enhances the Rate of Monolayer Formation as Assessed by Endothelial Cell Migration and Proliferation

**FIGURE 2:**
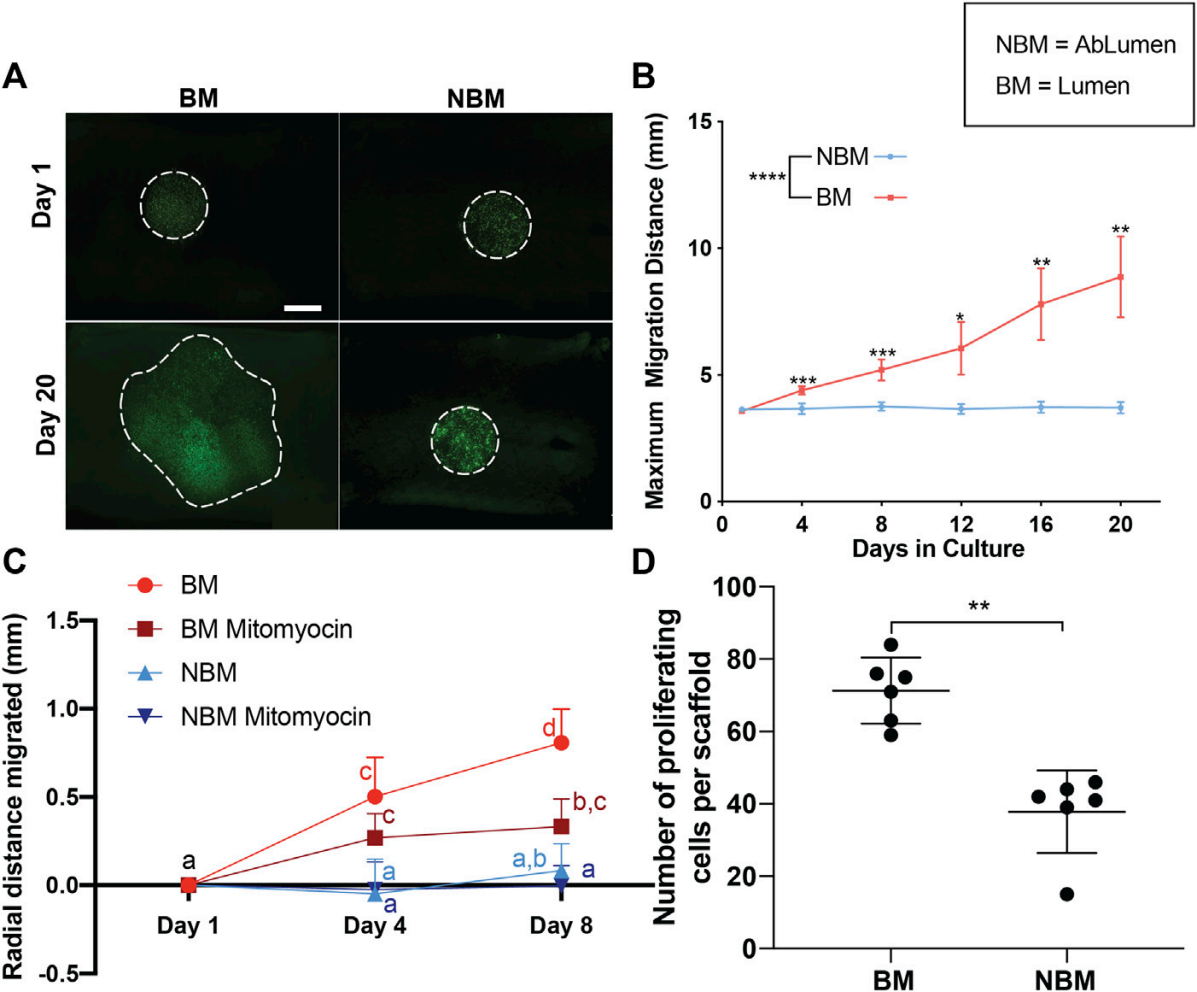
Effects of the presence (BM) versus the absence (NBM) of basement membrane proteins on human aortic endothelial cell (hAECs) migration and proliferation. Inverted microscopy images of GFP-labeled hAECs demonstrate that cells seeded on the BM surface cover a larger surface area than those seeded on the non-basement membrane protein (NBM) surface following 20 days in culture **(A)**. Dashed lines for Day 1 images indicate the initial seeding location of the cells. Dashed lines on Day 20 images indicate the final location of the cells. Due to the uneven surface of the scaffolds and the imaging method (stitch function in microscope Nikon Eclipse Ts2R), different values of cell brightness and focus are present in the images. A quantitative analysis of cell migration distance shows a significant increase in the maximal migration distance for cells seeded on the BM surface versus the NBM surface at all post-seeding timepoints **(B)**. Analysis of cell migration only (i.e., proliferation inhibited with mitomycin) shows a significant difference in the maximal radial distance migrated by treated-hAECs seeded on the BM surface (BM mitomyocin) when compared to treated-hAECs seeded on the NBM surface (NBM mitomyocin) at all timepoints **(C)**. Groups not connected by the same lower case letter are significantly different. Maximal migratory distance following 9 days of culture was reduced by the addition of mitomycin for cells seeded on the BM surface, but not for those seeded on the NBM surface **(D)** Assessment of proliferation only (i.e., FUCCI staining) demonstrated a significantly higher number of proliferating hAECs on the BM surface when compared to the cells in the NBM surface **(D)**. Scale bar A, 5,000 µm * = *p* < 0.05, ** = *p* < 0.01, *** = *p* < 0.001.

To determine the effect of basement membrane proteins on the rate of hAEC monolayer formation, proliferation, and migration were assessed for cells seeded in the presence (i.e., basement membrane surface—BM) or absence (i.e., non-basement membrane surface—NMB) of basement membrane proteins. To determine the effect on cell spread due to the combined effect of migration/proliferation, cells were seeded on either the BM or NBM surface of the AR-scaffolds in the same initial seeding diameter (i.e., 3.5 mm disc in the center of a 1 × 1 cm scaffold). While no differences were seen between the two sides at day 1, hAECs seeded on the BM surface covered a larger surface area than those seeded on the NBM surface by 20 days post-seeding (Figure 2A). A quantitative analysis of cell migration/proliferation demonstrated that the cells seeded on the BM surface spread farther each day [0.28 mm/day (0.13–0.37)] when compared to the cells seeded on the NBM surface [0.006 mm/day (0.008–0.01)] at all post-seeding time points [Figure 2B; day 4 (*p* < 0.001), day 8 (*p* < 0.001), day 12 (*p* < 0.05), day 16 (*p* < 0.01) and day 20 (*p* < 0.01)]. The effect of basement membrane presence vs the absence on hAEC migration alone was assessed by inhibiting the proliferation with mitomycin. Migration-only experiments demonstrated a significant difference in the maximal radial distance migrated by treated hAECs seeded on the BM surface (BM Mitomyocin, 0.043 mm/day [0.069–0.015]) when compared to treated hAECs seeded on the NBM surface (NBM Mitomyocin, 0.0032 mm/day [0.022–0.018]) at day 4 (*p* < 0.05) and day 8 (*p* < 0.05) post-seeding (Figure 2C). Inhibition of proliferation did not significantly reduce early (day 4) migration in the presence of basement membrane components. However, at later timepoints, proliferation inhibition decreased the maximal migratory distance (day 8) compared to combined migration/proliferation in the presence of basement membrane proteins. The effect of the basement membrane proteins’ presence vs absence on EC proliferation was assessed using FUCCI staining. Basement membrane presence (i.e., BM surface) resulted in a significantly higher number of proliferating hAECs [73 (62–78) per scaffold, *p* < 0.01] compared to the number of hAECs proliferating following seeding on the NBM surface [42 (Rieder et al., 2004; Gratzer et al., 2006; Jaffe et al., 2008; Martin-Belmonte and Mostov, 2008; Reing et al., 2009; Lampugnani et al., 2010; Bloch et al., 2012; Tousoulis et al., 2012; Lizama and Zovein, 2013; Yeong, 2013; Heo et al., 2015; Iorio et al., 2015; van Dijk et al., 2020) per scaffold, Figure 2D].

### 3.3 Presence of Basement Membrane Proteins Enhance the Endothelial Cell Quiescent Phenotype and Function in a Confluent Monolayer

**FIGURE 3:**
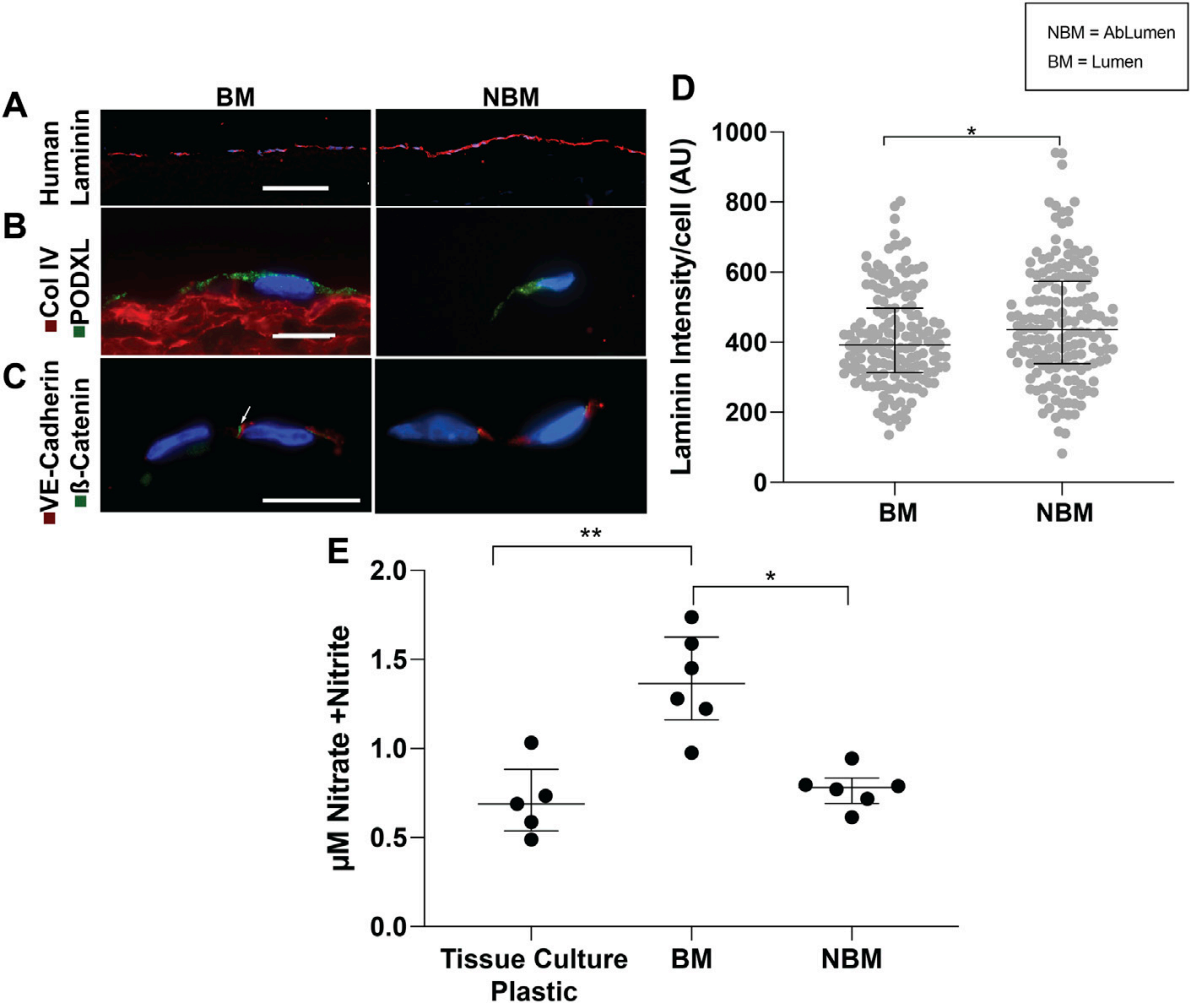
Effects of the presence (BM) versus the absence (NBM) of basement membrane proteins on hAEC phenotypes and functions. Representative images of immunofluorescent staining of human laminin production demonstrates the increased production of human laminin in the hAECs seeded on the non-basement membrane protein (NBM) surface when compared to cells seeded on the BM surface **(A)**. A quantitative analysis of the human laminin production per cell shows a significant higher production of human laminin by the hAECs seeded on the NBM surface versus on the BM surface **(D)**. Representative images of immunofluorescent staining for endothelial cell polarization demonstrates that the BM presence mediates the polarization of podocalyxin (green) to the apical cell surface of seeded hAECs, indicating their quiescent phenotype **(B)**. Conversely, apical polarization of podocalyxin is not present on hAECs seeded on the NBM protein side **(B)**. Similarly, representative images of the adherence junction immunofluorescent staining for VE-cadherin (red) and β-catenin (green) resulted in appropriate expression and co-localization of these adherence junction proteins (indicated by the arrow) in hAECs seeded on the BM surface, while expression and co-localization is not seen in cells seeded on the NBM protein surface **(C)**. A quantitative analysis of nitrate and nitrite secretions (nitric oxide biproducts) of HUVEC-seeded AR-scaffolds, show a significant increase in nitrate and nitrite secretions by cells seeded on the BM protein surface, compared to the cells seeded on the NBM protein surface or tissue culture plastic **(E)**. Scale bar A, 100 µm. Scale bars B and C, 10 µm * = *p* < 0.05, ** = *p* < 0.01.

The ability of basement membrane proteins in AR-SV scaffolds to drive the quiescent behavior of confluent endothelial cells (i.e., seeded as a monolayer) was determined using immunofluorescence and nitric oxide production. Qualitative assessment of human laminin production using immunofluorescence demonstrated that hAECs seeded on the BM side produced less human laminin that those seeded on the NBM surface (Figure 3A). Quantification of human laminin production per cell confirmed significantly lower human laminin production for cells seeded on BM surfaces [392 AU/cell (313.8–497), *p* < 0.05] compared to those on the NBM surface [435.9 AU/cell (338.8–574.2), Figure 3D]. Representative images of the immunofluorescence staining demonstrated apical polarization of podocalyxin (PODXL) in hAECs seeded on the BM surface, while polarization was absent in cells seeded on the NBM surface (Figure 3B). Similarly, the formation of adherence junctions was evident in hAECs seeded on the BM surface of AR-scaffolds, with appropriate co-localization of VE-cadherin and β-catenin. Conversely, adherence junction formation was lacking in the cells seeded on the NBM surface (Figure 3C). Finally., nitric oxide production of HUVECs was higher following BM surface seeding [1.36 µM Nitrate + Nitrite (1.16–1.63)] compared to the cells seeded on tissue culture plastic [0.64 µM Nitrate + Nitrite (0.31–0.81) *p* < 0.01] or the NBM surface [0.78 µM Nitrate + Nitrite (0.69–0.83), *p* < 0.05]. Nitric oxide production was not significantly different between cells seeded on tissue culture plastic and those seeded on the NBM surface (*p* > 0.05, Figure 3E).

### 3.4 Endothelial Cells Seeded as a Monolayer on the Basement Membrane Side of Antigen Removal-ECM Scaffolds Adopt a Transcriptional Phenotype Similar to Simvastatin Treated Human Aortic Endothelial Cells, but Distinct From TNF*α* Treated Human Aortic Endothelial Cells

**FIGURE 4:**
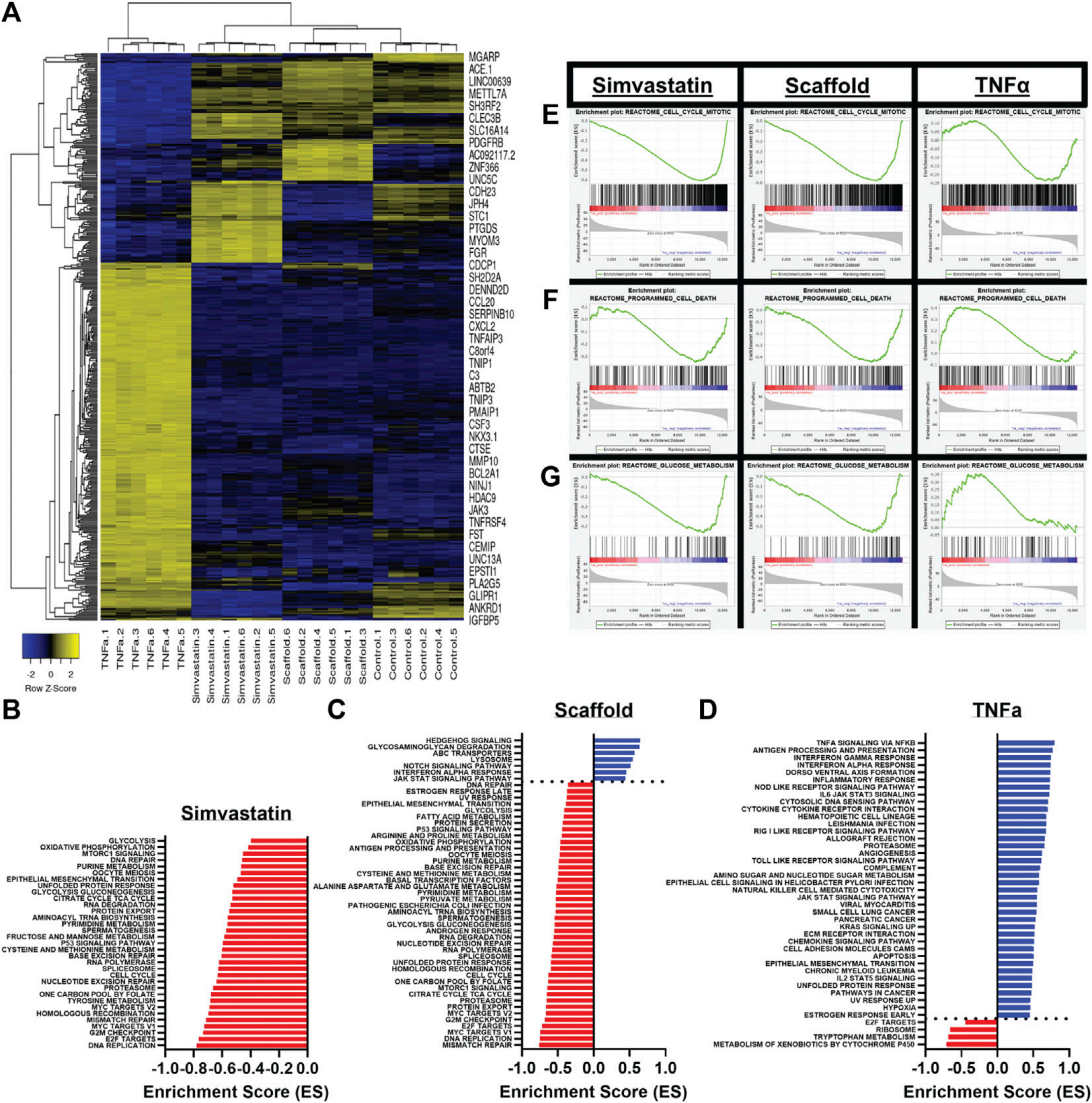
Differential gene expression and the gene set enrichment analysis of scaffold seeded, simvastatin-, and TNFα-treated hAECs. **(A)** Heatmap of all 415 identified significantly differentially expressed genes across all three treatment groups. **(B–D)** Significantly enriched pathways in the KEGG and HALLMARK gene sets following GSEA for simvastatin **(B)**, scaffold **(C)**, and TNFα **(D)** groups. **(E–G)** Representative GSEA plots across all three groups for three biologically relevant pathways, including mitosis **(E)**, programmed cell death **(F)**, and glucose metabolism **(G)**.

To determine the effect of BM presence on hAEC quiescence, cells seeded on AR-scaffolds were compared to quiescent (simvastatin) and activated (TNFα) hAECs *via* RNA sequencing. A total of 415 individual genes were determined to be significantly different in each of the three groups (scaffolds, simvastatin, and TNFα), compared to control. The TNFα group accounted for 306 of these differentially expressed genes, while the scaffold and simvastatin groups contained 59 and 85 differentially expressed genes, respectively. Only five genes (ACP5, CXCL8, FST, NOV, and ST6GALNAC1) were found to be differentially expressed across all three groups. Hierarchial clustering of the heatmap of all significantly differentially expressed genes across all three treatment groups demonstrated each respective group possessed its own distinct transcriptomic signature as evidenced by all six replicates within each treatment group clustering together (Figure 4A). Additionally, the scaffold group is seen clustered between the simvastatin and control groups, relatively removed from the TNFα group in the heatmap.

Gene Set Enrichment Analyses (GSEA) were employed to examine the pathways which were significantly enriched in each treatment group, compared to control. A total of 355 pathways (17 positive, 338 negative) were found to be significantly enriched in the scaffold- seeded hAECs from the REACTOME, HALLMARK, and KEGG gene set databases. Comparatively, the simvastatin group had a total of 279 (5 positive, 274 negative) and the TNFα group possessed a total of 133 (124 positive, nine negative) significantly enriched pathways. Bar plots showing the magnitude and directionality of significantly enriched HALLMARK and KEGG pathways for each of the three treatment groups indicated that the scaffold group (Figure 4C) was more similar to the simvastatin group (Figure 4B), than the TNFα group (Figure 4D) with respect to both the directionality and identity of the significantly enriched pathways. Representative GSEA plots for three biologically relevant pathways, including mitosis (Figure 4E), programmed cell death (Figure 4F), and glucose metabolism (Figure 4G) confirm this observation. For all three pathways shown, both the simvastatin and scaffold groups are negatively enriched, while the TNF*α* group demonstrates a positive enrichment.

### 3.5 tSNE Clustering Analysis Confirms the Similarity Between Scaffold-Seeded and Simvastatin-Treated Human Aortic Endothelial Cells, and Highlights the Disparity Between the TNF*α* Group

**FIGURE 5:**
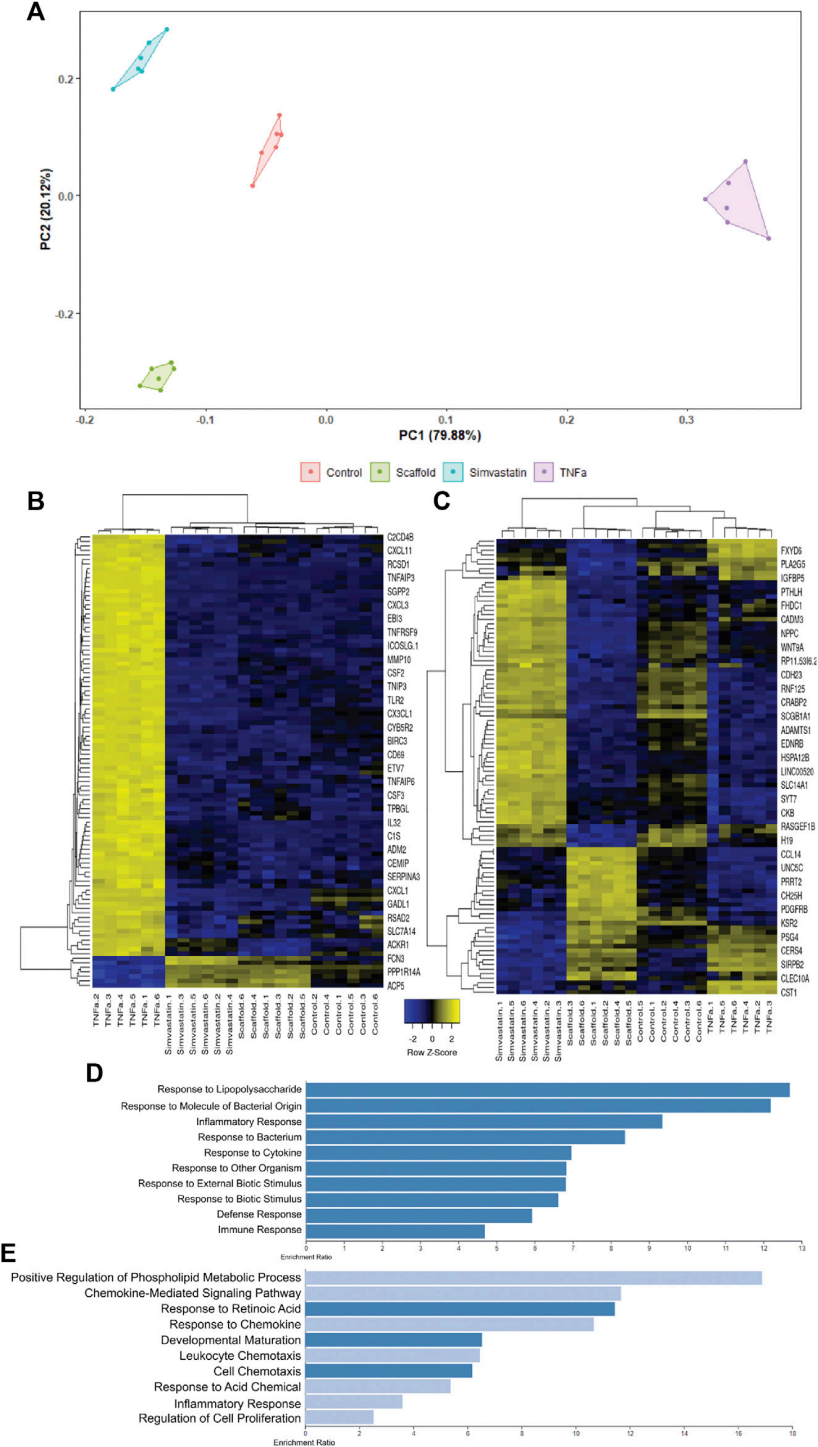
tSNE clustering analysis of scaffold seeded, simvastatin, and TNFα-treated hAECs. The tSNE clustering analysis plot is driven by the top 1,000 variable genes across all samples **(A)**. PC1 accounts for 79.88% of the variance, while PC2 explains the remaining 20.22%. Heatmap of the top 100 genes responsible for driving PC1 **(B)**. PC1 is almost exclusively driven by up-regulated genes in the TNFα group. Enrichment plot of the top 10 enriched gene ontology biological processes for the top 100 genes driving PC1 **(D)**. The top 10 enriched pathways in PC1 are all associated with pro-inflammatory responses. Heatmap of the top 100 genes responsible for driving PC2 **(C)**. PC2 is predominantly driven by up-regulated genes in the scaffold group. Enrichment plot of the top 10 enriched gene ontology biological processes for the top 100 genes driving PC2 **(E)**. 50% of the enriched pathways in PC2 are associated with cellular chemotaxis and respond to chemokines.

To further confirm hAECs seeded on the AR-scaffolds had a similar transcriptional profile to the simvastatin-treated cells, compared to the TNFα-treated cells; tSNE clustering analysis was performed based on the top 1,000 variable genes across all samples (Figure 5A). The Principal Component 1 (PC1), which accounts for 79.88% of the variance, shows a distinct separation between the TNFα-treated group and the other three groups (simvastatin, control, and scaffold). Interestingly, the Principal Component 2 (PC2), which explains the remaining 20.12% of the variance, highlights a separation between scaffold-seeded cells and the other three treatment groups, which were seeded on tissue culture plastic.

In order to better understand the differences between PC1 and PC2, the top 100 genes responsible for driving the differences observed in each Principal Component were extracted. Using the read count data, Z-scores for each of these genes were calculated and heatmaps were generated for both PC1 (Figure 5B) and PC2 (Figure 5C). PC1 is almost exclusively driven by up-regulated genes in the TNFα group (Figure 5B). When we performed an Over-Representation Analysis (ORA) on these genes using WebGestalt, the top ten enriched pathways generally reflected a pro-inflammatory response, of which, TNF*α* is a potent inducer (Figure 5D). Similar to what was observed in the tSNE clustering analysis, the heatmap for the top 100 genes in PC2 (Figure 5C) illustrates that there is a distinct difference between the cells seeded on the AR-ECM scaffold and cells seeded on tissue culture plastic (control, TNFα or simvastatin). Although there is not as distinct of a pattern in the ORA of the PC2 genes, as compared to the PC1 genes, of the top 10 enriched pathways, five (50%) pathways are related to cell chemotaxis and responding to chemokines (Figure 5E).

## 4 Discussion

The success of of-the-shelf small diameter vascular grafts is dependent on the ability of endothelial cells to rapidly form a monolayer on the luminal surface of the graft and to promote quiescent behavior once the monolayer has been established (Lopera Higuita and Griffiths, 2020b). Acellular ECM scaffolds derived from their target organs have gained importance in the field of tissue engineering due to their inherent characteristics. ECM proteins secreted by host cells are tailored for the specific needs and functions of each independent organ. In vascular physiology, basement membrane proteins are essential for vessel homeostasis due to their known individual roles in modulating endothelial cell behavior and vascular integrity. Consequently, basement membrane proteins may be expected to play a key role in promoting endothelialization and the resultant function of denuded ECM scaffolds when implanted as vascular grafts. The results in this article demonstrate that the AR process is capable of preserving the major components of native tissue basement membrane complex and the conjoint effect of these complexes modulates endothelial cell migration and proliferation, enhancing the rate of th monolayer formation and drives the quiescent behavior once a cell confluence has been achieved.

The importance of basement membrane proteins in vascular biology highlight the need for tissue-processing methods to retain the structure, composition, and function of this critical vascular component in acellular ECM scaffolds. Unfortunately, the goal of retaining the native vascular basement membrane integrity has proven challenging to achieve (Crapo et al., 2011). Previously reported de-cellularization methods have been shown to result in significant disruption of basement membrane integrity, although the extent to which each individual basement membrane protein is disrupted and/or eliminated varies depending on the de-cellularization process and specific tissue employed (Crapo et al., 2011). In the case of vascular grafts, de-cellularization methods have been universally reported to disrupt the delicate native BM components (Schaner et al., 2004; Crapo et al., 2011; Mancuso et al., 2014). Alternatively, antigen removal (AR) tissue-processing method has been designed to minimize macromolecule denaturation and disruption, while maximizing antigen solubilization and elimination, resulting in the production of ECM scaffolds with retained native ECM, including basement membrane proteins (Liu et al., 2016; Shklover et al., 2019; Xing et al., 2021). Indeed, in the current study, the capability of the AR tissue-processing method to retain both the composition and structural integrity of ECM and basement membrane proteins is clearly shown by the retention of appropriately oriented collagen, elastin, collagen IV (Col IV), and laminin. Consequently, unlike previously reported de-cellularization processes, the results presented here demonstrate the ability of the AR tissue-processing method to retain the integrity of the native small diameter vascular scaffold ECM components, including the delicate basement membrane complex (Bloch et al., 2012; Faulk et al., 2014b).

Basement membrane proteins are a specialized, thin layer of ECM proteins composed by a wide array of different proteins (Col IV, laminin, heparan sulfate proteoglycans, nidogen, fibrillin, collagen III, and osteonectin) that interact with each other to provide important structural and functional support to the overlying endothelial cells. Although being composed by multiple proteins, laminin and Col IV are among the few basement membrane proteins capable of modulating endothelial cell behavior *via* cellular receptors (Xing et al., 2021). In isolation, Col IV has been shown to facilitate endothelial cell migration and provide migration directionality (Heo et al., 2015). As predicted from single protein studies, the individual effects of Col IV persist in the present study. Endothelial cells seeded on the BM surface, and therefore exposed to Col IV, were able to migrate significantly farther than cells seeded on the NBM side. Although other literature in de-cellularized tissues has reported cell migration into ECM scaffolds, such studies failed to investigate whether a differential effect of the migration on the BM versus NBM environments existed (Rieder et al., 2004; Gratzer et al., 2006; Reing et al., 2009). Since alterations to the ECM proteins play a key role in decreasing cell chemotaxis, the aforementioned effect of de-cellularization on basement membrane integrity would be expected to abolish such differential cell migratory behavior (Rieder et al., 2004; Gratzer et al., 2006; Reing et al., 2009). Indeed, studies in vascular (i.e., saphenous vein) tissues support this contention, showing that destruction of the basement membrane environment by SDS-decellularization eliminates the effect of BM in driving rapid cell migration (Lopera Higuita and Griffiths, 2020a). The ability of the intact basement membrane of AR vascular scaffolds to increase endothelial cell migration in vascular grafts is an extremely desired scaffold attribute due to the absolute need for rapid endothelial cell monolayer formation to avoid scaffold failure *via* a wide array of mechanisms, including thrombosis (Lopera Higuita and Griffiths, 2020b).

Intriguingly, the absence of basement membrane proteins in the NBM surface also modulated differential cell behavior on the cells seeded on this surface. The cells seeded on the NBM surface initiated the over-expression and secretion of human laminin, a behavior that was less prominent in the cells seeded on the BM surface. The increased human laminin secretion can potentially be attributed to the exposure of endothelial cells to structural collagens (e.g. collagen I), to which they are only exposed during vessel injury. After vessel injury, the endothelial cells react by overexpressing basement membrane proteins, including laminin to provide the substrate for endothelial cell migration into the wound to re-establish an intact endothelial cell layer (Iorio et al., 2015). Furthermore, the confounded necessity to produce their own basement membrane proteins is the likely explanation for their decreased proliferative capacity. To undergo mitosis cells, in general, significantly down-regulate their protein trafficking, making it unviable for laminin over-expression and cell proliferation to happen at the same time (Yeong, 2013). Therefore, the need for basement membrane secretion by the endothelial cells seeded on the NBM surface of the scaffolds likely inhibited cell division, resulting in a lower number of proliferating cells.

In addition to promoting the rapid endothelial cell monolayer formation, an ideal vascular scaffold should also modulate quiescence of the re-populated cells once a confluent monolayer is achieved. Surfaces which uncontrollably drive endothelial cell activation result in aberrant endothelial cell signaling, proliferation and/or infiltration, leading to failure by intimal hyperplasia, altered vascular tone, recruitment of inflammatory cells, and/or permanently altered vessel wall structure (McKavanagh et al., 2017). Laminin, along with other basement membrane and ECM proteins, play an important role in driving endothelial cell maturation from actively growing cells toward a quiescent state (van Dijk et al., 2020). Consequently, laminin integrity is expected to be the key to avoid failure by modulating endothelial cell quiescence. Laminin availability to endothelial cells seeded on the BM surface of the scaffolds likely modulated the observed apicobasal polarization (i.e., podocalyxin covering the apical cell membrane only). Conversely, seeding in the NBM surface resulted in failure of quiescent apicobasal endothelial cell polarization. Apicobasal polarization of endothelial cells is an important characteristic of achieved quiescent state and reflects the capability of endothelial cells to maintain the stability and integrity of the vascular wall (Lizama and Zovein, 2013). Additionally, the appropriate formation of an apicobasal polarization is known to be an absolute requirement for correct lumen organization in any type of vessel (Jaffe et al., 2008; Martin-Belmonte and Mostov, 2008). Furthermore, the expression and formation of adherence junctions between the endothelial cells is an absolute pre-requisite to forming this apicobasal polarization (Lampugnani et al., 2010). Endothelial cell adherence junctions are protein complexes that form in the cell–cell junctions and function as sites of cell attachment, signaling, and regulation of growth and apoptosis, as well as vascular homeostasis. Basement membrane proteins (e.g., Laminin) present in the BM surface of the scaffolds provided the appropriate environment and signaling to modulate endothelial cell expression and formation of adherence junctions as indicated by appropriate VE-Cadherin and β-catenin co-localization. Conversely, the lack of BM proteins in the NMB surface resulted in the lack of formation of adherence junctions and the resultant lack of apicobasal polarization. Failure to maintain apicobasal polarization is known to be associated with decreased Col IV and laminin production resulting in aneurysmal dilation and increased vascular permeability (Lampugnani et al., 2010). Finally, additional evidence of the quiescent endothelial cell phenotype for cells seeded on the BM surface was observed due to their capacity to secrete nitric oxide (NO) in higher concentrations than those cells seeded on the NBM surface or tissue culture plastic. NO is a soluble gas that aids in the control of a wide array of biological processes key for vessel homeostasis and normal endothelial cell function (Tousoulis et al., 2012). Its role in preventing vascular diseases is known to be so essential that significant efforts have been allocated into engineering vascular grafts with the capability of synthetically producing and/or releasing NO (Wang Z et al., 2015; Wang Y et al., 2015). Therefore, the capability of AR-scaffolds to naturally modulate endothelial cells to secrete NO is an important achievement toward the development of a small diameter vascular graft. Collectively, the ability of the intact basement membrane of AR vascular scaffolds to drive apicobasal polarization, adherence junction formation, and NO production demonstrate the capacity of such scaffold environments to promote endothelial cell quiescence.

Finally, in order to conclusively determine that the intact basement membrane of AR vascular scaffolds promotes endothelial cell quiescence in an unbiased manner, RNA-sequencing was performed to compare the transcriptome of scaffold-seeded cells to the transcriptomes of endothelial cells treated with either simvastatin or TNFα. Simvastatin, a member of the statin drug family, commonly prescribed to patients as a means to lower their cholesterol levels. However *in vitro*, simvastatin has previously been shown to improve endothelial cell function and potentially induce physiologic changes which reduce the risk of thrombosis development (Beckman and Creager, 2006). Conversely, TNFα is a potent pro-inflammatory cytokine, well known to modulate proliferative and inflammatory behaviors in endothelial cells (Rastogi et al., 2012). In this manner, a continuum between cellular quiescence and cellular activation was established. This was utilized to compare the transcriptional profile of endothelial cells seeded on the BM surface of AR-ECM venous scaffolds and determine how the BM surface influenced the cellular phenotype. It is important to point out that RNA sequencing for cells seeded on the NBM side of the scaffolds could not be performed due to the inability to extract non-fractioned RNA from cells when seeded on this surface. Following the bioinformatics analysis, several pieces of evidence were identified which confirmed that endothelial cells seeded on scaffolds adopted a phenotype more closely resembling simvastatin-treated, rather than TNFα-treated cells. Specifically, the unsupervised hierarchial clustering based on all identified significantly differentially expressed genes across all three treatment groups indicated that the scaffold samples had a similar transcriptional signature to the simvastatin samples, and a signature distinct from the TNFα group (Figure 4A). Furthermore, when compared head-to-head, gene set enrichment analyses for each of the three treatment groups indicated that scaffold-seeded cells had a similar magnitude and directionality of enrichment for various pathways as the simvastatin group, but opposite to that of the TNF*α* group. Additionally, many of the commonly enriched pathways between the simvastatin and scaffold groups were related to processes that are commonly down-regulated in quiescent cells. In particular, there was a strong negative enrichment in pathways relating to the cell cycle, programmed cell death, and metabolism in these two groups (Cho et al., 2019; Li et al., 2019). Conversely, however, these processes were positively enriched in the endothelial cells treated with TNFα (Figures 4E–G), consistent with the up-regulation of these processes upon cellular activation. Furthermore, tSNE clustering analyses demonstrated distinct differences between the TNFα-treated cells and the other three groups (as evidenced by PC1, which accounted for ∼80% of the variance). The main component driving the variance in PC1 is a strong up-regulation of the genes in the TNFα group, which are not up-regulated in the other three groups. An over-representation analysis (ORA) of these PC1 genes confirms this finding, as all 10 of the top 10 most enriched gene ontology-biological processes pathways are pro-inflammatory/regulated by TNF*α* (Figure 5C). Interestingly, PC2 (which explained the remaining ∼20% of the variance) showed a separation between the three groups seeded on tissue culture plastic and the cells which were interacting with the BM of AR vascular scaffolds (Figure 5A). Additionally, as suspected based on the tSNE clustering analysis, PC2 appears to be driven primarily between differences between the scaffold group and the other three groups which were seeded on tissue culture plastic (Figure 5D). In the same manner, an ORA of these genes indicated that half of the top ten (5/10) most enriched pathways were related to cellular chemotaxis/response to chemokines (Figure 5E). It is well established that the extracellular matrix is a reservoir for numerous cell signaling molecules which can modulate cell behaviors (Valiente-Alandi et al., 2016; Poltavets et al., 2018). The fact that cells interacting with AR ECM scaffolds would be enriched for pathways related to chemotaxis further demonstrates that the seeded endothelial cells are interacting in appropriate biologically relevant ways to the active components of ECM. Overall, these results indicate that hAECs seeded on the BM (luminal) side of AR ECM venous scaffolds respond to inherent ECM signals, mediating the observed cellular migration, proliferation, and quiescent phenotype.

## 5 Conclusion

The results in this manuscript indicate that the intact basement membrane proteins present in AR vascular scaffolds positively modulate a wide array of endothelial cellular processes resulting in the rapid re-cellularization and quiescent endothelial cell monolayer formation, which is known to be essential for vascular homeostasis. Therefore, we conclude that the retention of basement membrane proteins by tissue processing methods is essential for the success of small diameter vascular grafts. Due to the capability of AR to retain the macromolecular structure, organization, and composition of the proteins within the ECM scaffold, future work is required to separate the individual effects of each of the basement membrane protein’s macromolecular integrity and structure of the resultant surface on resultant endothelial cell responses.

## Data Availability

The dataset presented in this study can be found in online at GEO, accession number GSE199281,https://www.ncbi.nlm.nih.gov/geo/query/acc.cgi?acc=GSE199281.
