# Supplementary material for: Basement Membrane of Tissue Engineered Extracellular Matrix Scaffolds Modulates Rapid Human Endothelial Cell Recellularization and Promote Quiescent Behavior After Monolayer Formation
Source: Front Bioeng Biotechnol. 2022 Aug 2;10:903907. doi: 10.3389/fbioe.2022.903907 (PMC9379346; doi:10.3389/fbioe.2022.903907)
Supplement: Supplementary file 1 [file DataSheet1.PDF]

Supplemental Material

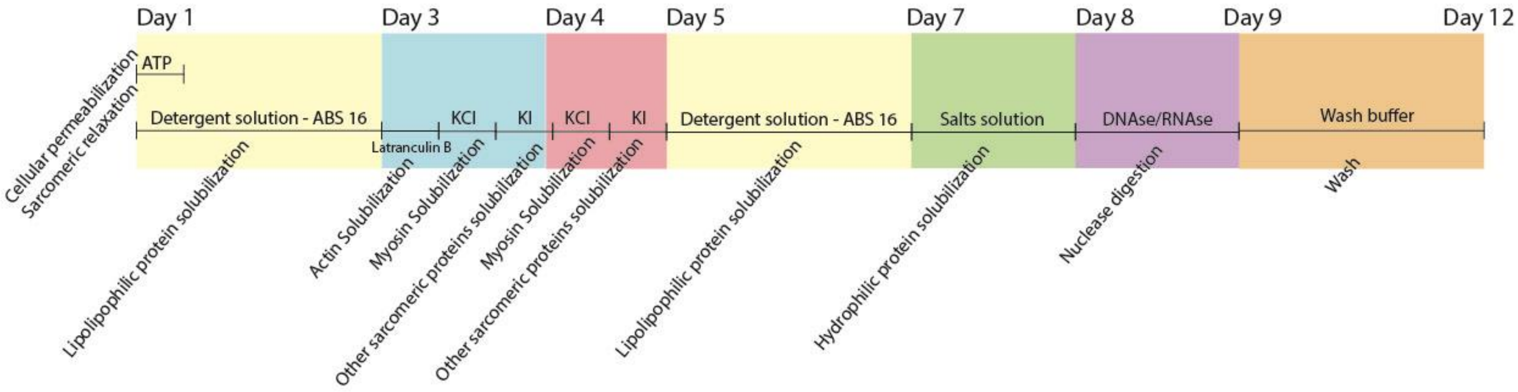

Supplemental Figure 1: Schematic of the Antigen Removal process. Cellular permeabilization and sarcomeric relaxation were achieved by incubation in lipophilic protein solubilization and Na-ATP two times for 30 min each. Relaxed veins were incubated in lipophilic protein solubilization solution for 48 h, followed by sarcomeric disassembly via incubation in Latranculin B for 2 h, 0.6 M potassium chloride (KCl) for 2 h and potassium iodine (KI) for 2 h. The KCl and KI steps were repeated the next day, followed incubation in lipophilic protein solubilization solution another for 48 h and incubation in hydrophilic protein removal solution for 24 h. Finally, samples were incubated in nuclease digestion for 24 h and washout of 96 h.
